# Herding-like behaviour in medical decision making: An experimental study investigating general practitioners’ prescription behaviour

**DOI:** 10.1371/journal.pone.0297019

**Published:** 2024-07-08

**Authors:** Sandro Tiziano Stoffel, Rachel Spencer, Judit Konya, Ivo Vlaev, Matthias Schwenkglenks

**Affiliations:** 1 Research Department of Behavioural Science and Health, UCL, London, United Kingdom; 2 Institute of Pharmaceutical Medicine (ECPM), University of Basel, Basel, Switzerland; 3 Department of Public Health, Health Economics Facility, University of Basel, Basel, Switzerland; 4 Unit of Academic Primary Care, Warwick Medical School, University of Warwick, Coventry, United Kingdom; 5 Exeter Collaboration for Academic Primary Care (APEx), University of Exeter, Exeter, United Kingdom; 6 Warwick Business School, University of Warwick, Coventry, United Kingdom; Eduardo Mondlane University: Universidade Eduardo Mondlane, MOZAMBIQUE

## Abstract

Previous observational studies have indicated that social influences, such as arising from herding-like behaviour, can contribute to medical errors. In this study, we experimentally examined whether general practitioners (GPs) would follow incorrect prescription recommendations from fellow GP or specialists. To investigate this, we conducted an online survey with 475 GPs practicing in England that included two case vignettes. Case vignette 1 focused on sleeping tablets, and case vignette 2 was centred around antibiotics. The vignettes were presented in random order, and within each vignette, study participants were assigned to one of three experimental conditions: *control condition* (lacking peer recommendation), *fellow GP condition* (including a recommendation from a fellow GP not aligned with best practice clinical guidelines), or *specialist condition* (including a recommendation from a specialist not aligned with best practice clinical guidelines). The primary outcome measure was the proportion of GPs who prescribed medication that deviated from best practice clinical guidelines. We found that, in both case vignettes, the percentage of respondents prescribing such medication was highest in those assigned to the *specialist condition*, followed by those assigned to the *control condition*. It was lowest in those assigned to the fellow *GP condition* (case vignette 1: 73.8% vs. 55.6% vs. 36.6% and case vignette 2: 24.0% vs. 12.4% vs. 10.1%). In the case of vignette 1, the difference between the *fellow GP condition* and the *control condition* is statistically significant, suggesting that GPs are less likely to prescribe sleeping tablets when recommended by a fellow GP. This implies that GPs are more inclined to prescribe non-guideline-recommended medication when advised by specialists. This study is the first to experimentally demonstrate that physician herding behaviour can result in prescription errors. Future research could extend this inquiry to diverse contexts, including diagnosis.

## Introduction

The majority of individuals who interact with healthcare providers typically receive high-quality care. However, some may encounter medical errors, such as incorrect diagnoses, treatments, and prescriptions, which can be detrimental to patients and result in significant financial consequences for health systems [1–3]. Systematic literature reviews have revealed the frequent occurrence of medical errors across various healthcare settings, including prescribing and prescription errors [4,5]. These errors can account for up to 11% of all prescriptions, with dosage errors being a primary concern [5,6]. Prescription-making is a complex task influenced by various factors, including physicians’ therapeutic training, drug knowledge, experience, understanding of the patient, perception of risk, and their own physical and emotional well-being [7,8]. While unsurprisingly, especially newly graduated physicians are prone to making prescription errors, senior doctors make errors too, albeit at a lower rate [9–11]. Recent studies have suggested that social influences, including social norms [12], informational social influence [13], and compliance [14], can influence physicians’ decision-making, potentially resulting in prescription errors [15]. These influences may lead to herding-like behaviour, where physicians follow recommendations from colleagues rather than making their own informed decisions based on available information. In some cases, this behaviour can lead to physicians adopting wrong recommendations. Social influences have been vastly studied in the context of stock markets [16–20]. There experiments have shown that even in the ideal environment of perfect knowledge, the study participants followed the decision of others, creating bubbles and market crashes [19]. Herding behaviour has been described as response to uncertainty [20].

So far little is known about herding-like behaviour in medical decision making. Only three non-experimental studies investigated the social influence of peers on physicians’ decision making [15,21,22]. The first study used a cross-sectional survey to investigate the prevalence and associated factors of social influences in multiple sclerosis (MS) care in Spain [15]. In the survey, neurologists with expertise in MS care were presented with a case vignette that featured a treatment recommendation of a peer, which was not supported by best practice clinical guidelines. Note that while the authors suggest that around 78% of the neurologists showed herding-like behaviour, in that they followed the wrong recommendation, the study did not feature a control condition without a treatment recommendation. In multivariate analyses, experience in the form of the number of patients seen per week was found to be positively associated with herding-like behaviour. Neurologists’ personality traits, such as risk aversion, aversion to ambiguity, and low tolerance to uncertainty, however, showed no association with herding-like behaviour.

The second study utilized longitudinal data on prescribed antipsychotic drugs for schizophrenia patients in Taiwan and found that physicians’ prescriptions of newly introduced drugs were influenced by the observed behaviour of their colleagues [21]. Peer influence on acceptance of a risky new prescription drug was similarly identified by Iyengar and colleagues [22] in an extensive survey. In their study, peer influence had an impact on both initial trial prescriptions and subsequent repeat orders for a novel and risky drug, particularly among physicians who did not view themselves as opinion leaders.

## Study overview, objectives and hypothesis

Despite this observational evidence indicating social influences on physicians’ decision-making, there is a lack of experimental studies comparing decision-making with and without input from peers. In the current study, we conducted an experiment to determine whether general practitioners (GPs) follow prescription recommendations made by other medical professionals. Specifically, we employed a randomised, web-based behavioural experiment to assess whether incorrect recommendations from other physicians could lead to potential prescription errors in a primary care setting, which is a highly relevant area. In England, the cost of medicines prescribed in primary care in 2022 and 2023 was £9.59 billion, 50.0% of total expenditure [23]. To prescribe medications optimally GPs must assess the appropriateness of medication for each patient, including considerations such as dosage, frequency, and type of medication to be used [24]. Our study seeks to address the following research question (RQ):

**RQ:** To what extent do GPs follow prescription recommendations of other physicians?

### Methods

To address this research question, we formulated three working hypotheses (WH). The first hypothesis builds on the focus theory of normative conduct [25] and assumes that the GP or consultant’s recommendation is a social norm of the descriptive type that refers to perceptions of how others would behave in this situation [26]. Focus theory predicts that the peer’s recommendation provides the decision-makers with a standard from which they do not want to deviate in the given situation [27]. By simply registering what others have done in the same situation and imitating their actions, one can usually choose efficiently and well how to behave [26]. Thus, rather than making their own informed decision based on available information, GPs will follow the prescription recommendation of other medical professionals.

**WH1:** GPs in the treatment conditions are more likely to select the prescription option that is not supported by best practice clinical guidelines than those in the control condition.

The second hypothesis addresses that GPs may believe the expertise of specialists is better than that of fellow GPs, as specialists are senior physicians, trained and certified in specific areas of medical practice.

**WH2:** A recommendation from a specialist will further increase the likelihood of selecting the prescription that is not supported by best practice clinical guidelines than a recommendation from a fellow GP.

The third hypothesis assumes, in line with previous studies on reputation-based herding, that the herding-like behaviour is accentuated in GPs with more work experience [15,28].

**WH3:** GP’s working experience is positively correlated with herding-like behaviour.

We tested the hypotheses through a randomized online experiment involving General Practitioners (GPs). The experiment was run in the first week of July 2023 and utilized a three-condition between-subjects design, comprising a control condition without a recommendation from a peer and two treatment conditions. Both treatment conditions involved a prescription recommendation that is not supported by best practice clinical guidelines. In the first condition, the recommendation originated from a fellow GP, while the second treatment condition included a recommendation from a specialist (geriatrician/respiratory consultant).

### Ethical approval and preregistration

The protocols for the study received ethics approval from the Humanities and Social Sciences Research Ethics Committee (HSSREC) of University Warwick (approval number HSSREC 155/22-23) and was preregistered on Open Science Framework before data collection.

### Vignettes

In order to test the working hypotheses, we conducted a web-based experiment that presented GPs two case vignettes in random order (screenshots of the vignettes are presented in S1 and S2 Figs). After consenting to participate in the study, participants were asked some basic questions about their work as GP, including the number of patients they see, their work experience, and the number of other GPs practicing in their clinic. They were then presented with two case vignettes, which were adapted by medical experts from previous literature and tested in pilot studies with experts in the field of academic primary care as well as practicing primary care physicians before the study was conducted. One case vignette described a situation of potentially inappropriate prescribing of sleeping tablets to a 70-year-old patient who was having trouble sleeping [29]. The second case vignette focused on antibiotic prescription and described a 27-year-old woman with no known underlying lung disease. She presented with a 10-day history of cough, producing yellow non-bloody sputum [30]. For each case vignette, participants had to select one of two prescription options, of which one was supported by best practice clinical guidelines (no medication) and the other was not (sleeping tablets in the first case vignette and antibiotics in the second case vignette)

### Outcome measures

The primary outcome of the study was the proportion of GPs who prescribed the medication. Specifically, participants were asked which of the two available prescriptions they would recommend in each vignette, 1) the prescription of a sleeping tablet/antibiotics or 2) no medication. Both prescription options were presented in random order. For each vignette, we compared the proportion of participants who recommended the incorrect prescription across the experimental conditions.

Secondary outcomes included findings on perceived decision difficulty and decision effort. Study participants were asked after each vignette how difficult it was for them to decide, using a fully labelled five-point Likert scale (‘Not at all’, ‘Slightly’, ‘Moderately’, ‘Very’ and ‘Extremely’) in response to the question ‘How difficult was it for you to make the recommendation?’ They were also asked to indicate their decision effort on a similar fully labelled five-point Likert scale (‘None’, ‘Little’, ‘Some’, ‘Considerate’, and ‘Great’) for the question ‘How much effort did you put into making the recommendation?’ Both questions were adapted and simplified from a 12-item subjective measurement of mental load and mental effort [31].

Additionally, we included ten questions from the decision style scale (DSS), which capture a broad range of rational and intuitive styles [32]. The scale contains five items on rational and five on intuitive dimensions. The response options featured fully labelled five-point ratings Likert scales (1 = strongly disagree to 5 = strongly agree). The global scores of the two decision styles are calculated as the sum of the corresponding five items and range between 5 and 25.

Finally, individual risk preferences were assessed by asking: “*Generally*, *in the different domains of your daily life*, *would you describe yourself as someone who tries to avoid risks (risk-averse) or as someone who is fully prepared to take risks (risk-prone)*. *Please answer on a scale from 0 to 10*, *where 0 means “risk-averse” and 10 means “risk-prone”*. The question was adapted from previous studies [33,34].

### Statistical analysis

To test the first two working hypotheses regarding the influence of peer recommendations on the proportion of participants prescribing a medication, we employed Chi-square tests of independence and binary logistic regressions. These analyses were adjusted for experimental conditions, decision style, risk preferences, and sociodemographic variables. Regarding the third working hypothesis, we used binary logistic regressions with an interaction term between experimental condition and work experience. The impact on perceived decision difficulty and cognitive effort was analysed using unadjusted and adjusted ordered logistic regressions. Additionally, we used Chi-square tests of independence, Fisher’s exact test, and ANOVAs to compare the characteristics of the study participants across the experimental conditions. Bonferroni adjusted significance levels were used for multiple comparisons between the three experimental conditions.

### Sample size

The sample size was calculated before data collection using estimates from previous studies for the control condition [29,30]. A sample size calculation suggested minimum group sizes of 150 participants per experimental condition to be able to detect differences of at least 17% in the proportion of participants choosing to prescribe medication between two conditions [35]).

## Results

### Study participants

**Fig 1:**
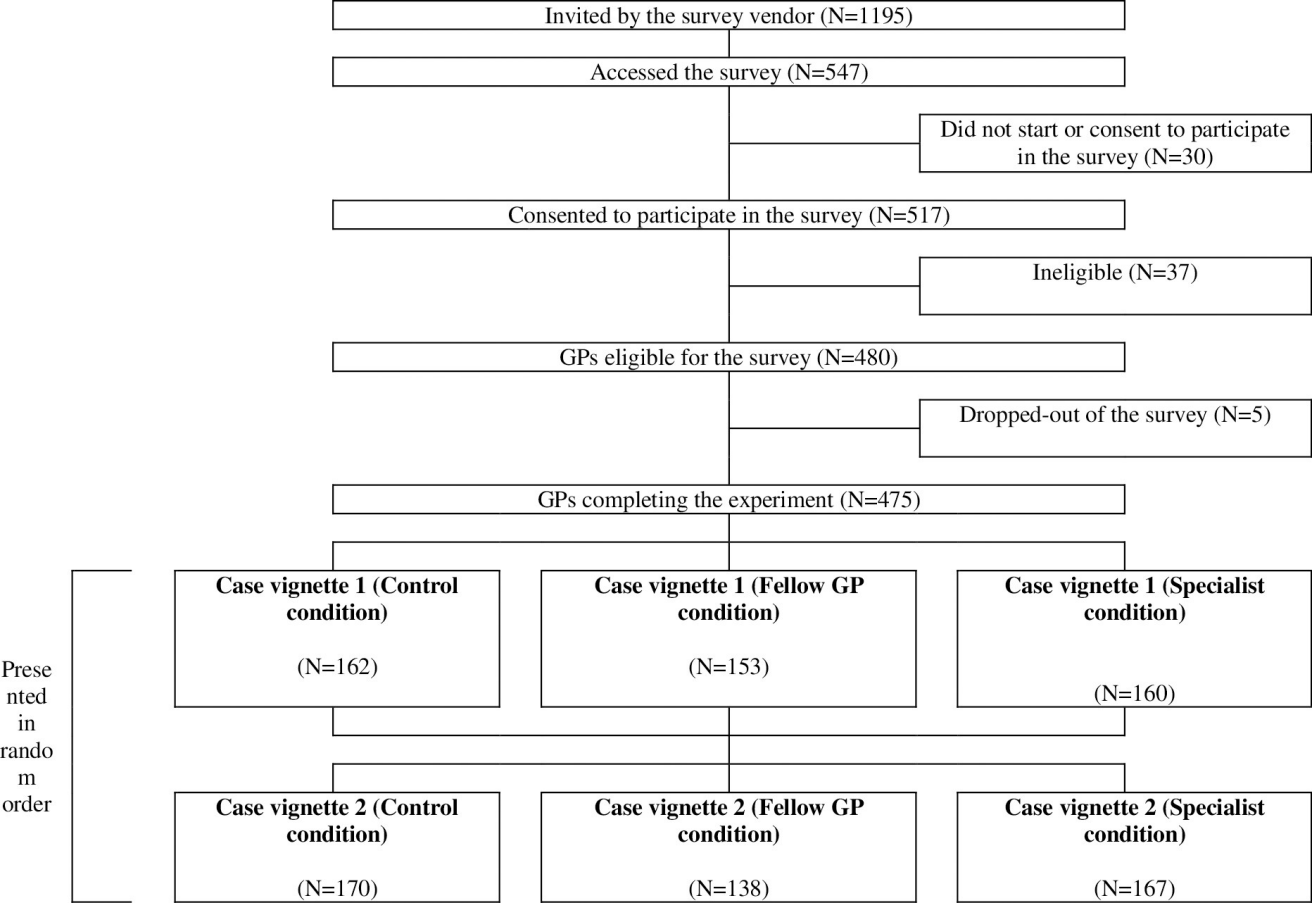
Flow through the study.

Fig 1 depicts the flow of the survey. In total, 1195 GPs practicing in England and registered on a survey panel (M3 Global Research) received invitations to participate in the study. Among the 547 (45.8%) who accessed the survey, 517 (94.5%) provided their consent to participate. A total of 37 study participants (7.2%) were excluded due to not having worked as a general practitioner for at least 2 years, and 5 respondents (1.0%) did not complete the survey, resulting in a final sample of 475 responders.

The characteristics of the participating GPs are summarized in Table 1. The most frequent categories among respondents were as follows: up to 39 years old (44.0%), female (54.1%), with 11 to 20 years of work experience (34.3%), practicing in a facility with 6 to 10 practitioners (41.7%), and serving more than 10,000 patients (54.1%). The distribution of the risk preferences, rational decision-making score and intuitive decision-making score are presented in S3–S5 Figs. On average, participating GPs exhibited tendencies toward risk aversion (Mean 4.34, Standard Deviation (SD) 2.00) and displayed a rational decision-making style (Mean 21.12, SD 2.52), with only a somewhat intuitive approach (Mean 14.25, SD 3.24). The characteristics were balanced across the three experimental conditions in the two case vignettes (see S1 and S2 Tables).

**Table 1:** Description of study sample (N = 475).

Variable	N	(%)
Age		
Up to 39 years	209	(44.0)
Between 40 and 49 years	178	(37.5)
Between 50 and 59 years	64	(13.5)
60 years or older	24	(5.0)
Gender		
Female	257	(54.1)
Male	213	(44.8)
Other	5	(1.1)
Work experience		
Between 2 and 5 years	105	(22.1)
Between 6 and 10 years	136	(28.6)
Between 11 and 20 years	163	(34.3)
More than 20 years	71	(15.0)
Number of GPs working in the practice		
Just me	3	(0.6)
Between 2 and 5	179	(35.8)
Between 6 and 10	198	(41.7)
More than 10	104	(21.9)
Number of patients registered in the practice		
Up to 1000	10	(2.1)
Between 1001 and 5000	44	(9.3)
Between 5001 and 10000	164	(34.5)
More than 10000	257	(54.1)
Region in which GP practices		
London	110	(23.2)
West Midlands	59	(12.4)
East Midlands	49	(10.3)
South West	45	(9.5)
South East	78	(16.4)
Yorkshire and the Humber	43	(9.0)
North West	19	(4.0)
North East	72	15.2)
Risk preference [1;10]–Mean and standard deviation	4.34	(2.00)
Rational decision making [5;25]–Mean and standard deviation	21.12	(2.52)
Intuitive decision making [5;25]–Mean and standard deviation	14.25	(3.24)

### Effect on prescription behaviour

**Fig 2:**
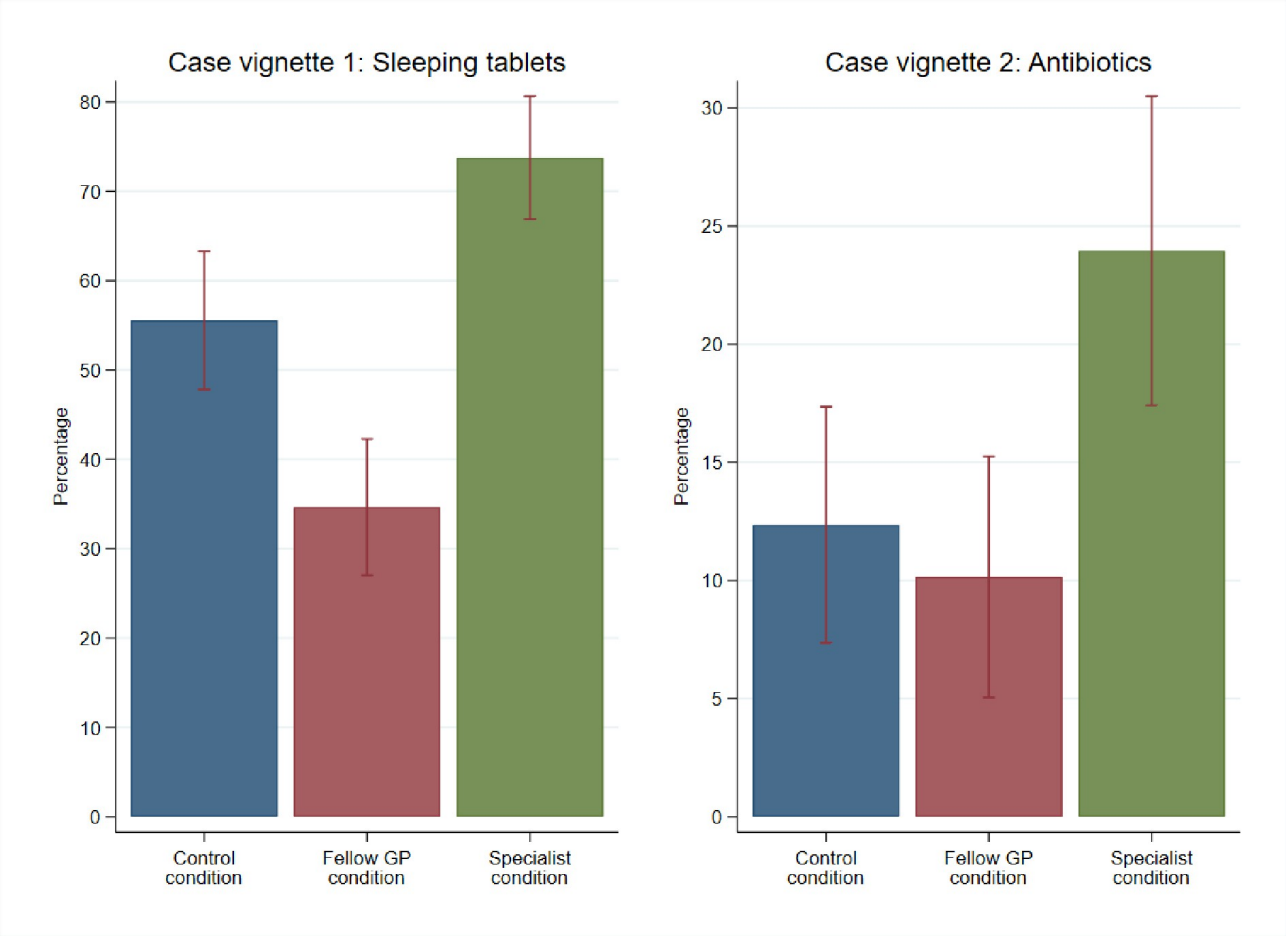
Prescribing medication in the two case vignettes.

In line with working hypothesis 2, Table 2 and Fig 2 indicate that GPs were more inclined to prescribe a medication when they received a comparable recommendation from a specialist. In both case vignettes, the percentage of respondents who prescribed the medication was highest among those exposed to the specialist condition, followed by individuals in the control condition, and finally, those in the fellow GP condition (case vignette 1: 73.8% vs. 55.6% vs. 36.6%, χ2(2, N = 475) = 48.37, p<0.001 and case vignette 2: 24.0% vs. 12.4% vs. 10.1%, χ2(2, N = 475) = 13.19, p = 0.001).

**Table 2:** Binary logistic regressions on prescribing a medication that deviates from best practice clinical guidelines in the two case vignettes.

	Share of GPs prescribing (%)	Sleeping tablets				Share of GPs prescribing (%)	Antibiotics			
		Unadjusted model		Adjusted model^a^			Unadjusted model		Adjusted model^a^	
		OR	95% CI	aOR	95% CI		OR	95% CI	aOR	95% CI
Overall	(55.0)					(15.8)				
Condition										
Control	(55.6)	Ref.		Ref.		(12.4)	Ref.		Ref.	
Fellow GP	(36.6)	0.424	0.269–0.668**	0.416	0.256–0.676**	(10.1)	0.801	0.391–1.641	0.885	0.417–1.881
Specialist	(73.8)	2.248	1.406–3.593**	2.168	1.325–3.546**	(24.0)	2.235	1.253–3.986**	2.409	1.298–4.472**
N		475		475			475		475	

* *p*<0.05

** *p*<0.01.

a Covariates included in the adjusted models are GP’s age, gender, work experience, number of GP working in the practice, number of patients, region in which GP practices, risk preferences, rational decision-making score and intuitive decision-making score. The full models are presented in the S3 and S4 Tables.

The adjusted regressions confirmed that receiving a recommendation from a specialist increased the probability of prescribing sleeping tablets (adjusted odds ratio (aOR) 2.17, 95% Confidence Interval (CI): 1.33–3.55, p = 0.002) and antibiotics (aOR 2.40, 95%CI: 1.30–4.47, p = 0.005). The recommendation of the fellow GP only influenced the prescription decision in the sleeping tablet vignette (aOR 0.46 95%CI: 0.26–0.68, p<0.001) but not in the antibiotics vignette (aOR 0.89 95%CI: 0.42–1.88, p = 0.751).

We found limited evidence for the third working hypothesis (see S4 Fig). The regression models for the sleeping tablet case vignette, which incorporated an interaction term for working experience and experimental condition, demonstrated similar patterns for GPs with both less than 10 years of experience and those with more than 10 years (see S5 and S6 Tables and S6 Fig). In both groups, the highest percentage of respondents prescribed the medication in the specialist condition, followed by the control condition, and then the fellow GP condition (up to 10 years: 71.1% vs. 55.8% vs. 30.6%, χ2(2, N = 241) = 25.74, p<0.001; and more than 10 years: 76.6% vs. 55.3% vs. 38.3%, χ2(2, N = 223) = 23.67, p<0.001). While the patterns were similar in the antibiotics case vignette, there was only a statistically significant influence of the recommendations on the prescription decisions in those with more than 10 years of work experience (up to 10 years: 18.2% vs. 13.7% vs. 5.8%, χ2(2, N = 241) = 5.07, p = 0.079; and more than 10 years: 28.9% vs. 10.7% vs. 14.5%, χ2(2, N = 223) = 10.09, p = 0.006)

### Effect on perceived decision difficulty and decision effort

**Fig 3:**
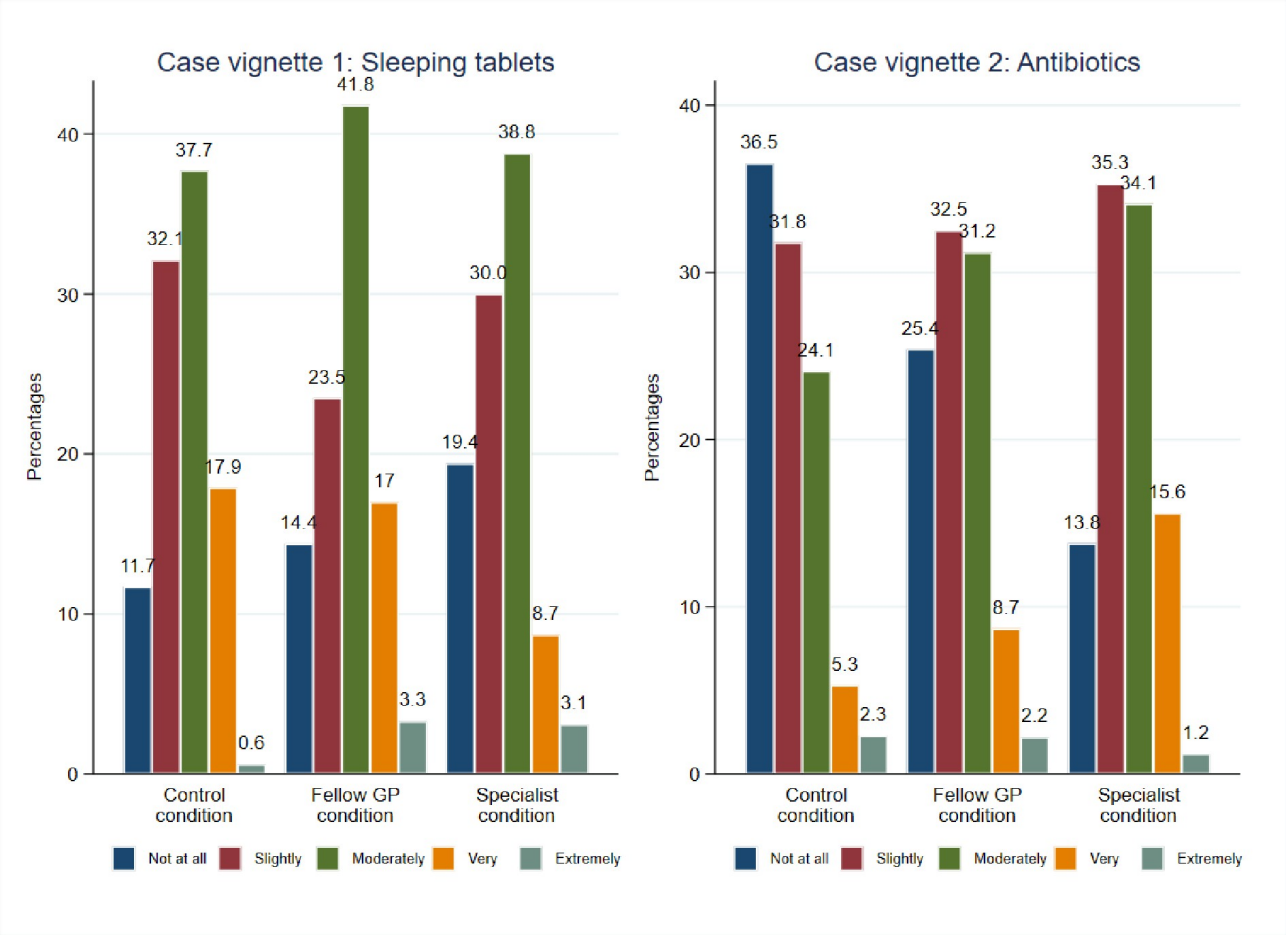
Perceived decision difficulty regarding the prescription decisions in the two case vignettes [1;5].

Fig 3 shows the perceived decision difficulty in the two case vignettes.

The ordered logistic regressions, as presented in Table 3, indicate that recommendations from peers led to an increase in the perceived decision difficulty in the antibiotics case vignette (fellow GP vs control: aOR 1.55 95%CI: 1.00–2.39, p = 0.046, specialist vs: control condition: aOR 2.64 95%CI: 1.75–3.98, p<0.001).

**Table 3:** Ordered logistic regression on perceived decision difficulty [1;5].

	Sleeping tablets				Antibiotics			
	Unadjusted model		Adjusted model*		Unadjusted model		Adjusted model*	
	OR	95% CI	aOR	95% CI	OR	95% CI	aOR	95% CI
Overall								
Condition								
Control	Ref.		Ref.		Ref.		Ref.	
Fellow GP	1.159	0.775–1.734	0.416	0.256–0.676**	1.632	1.080–2.467*	1.552	1.007–2.391*
Specialist	0.712	0.479–1.058	2.168	1.325–3.546**	2.607	1.757–3.867**	2.642	1.754–3.978**
N	475		475		475		475	

* *p*<0.05

**Fig 4:**
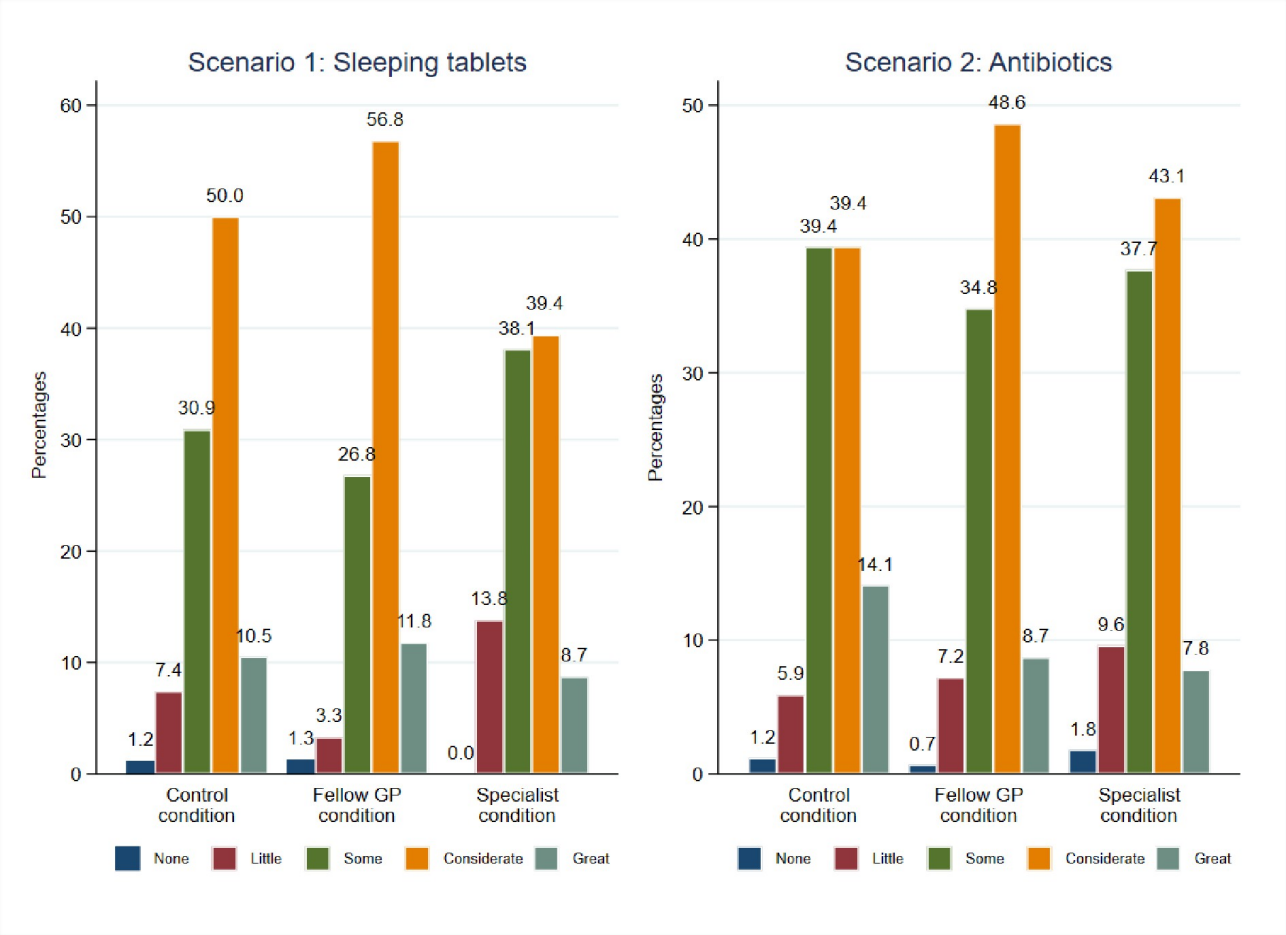
Decision effort in the two case vignettes [1;5].

** *p*<0.01.

a Covariates included in the adjusted models are GP’s age, gender, work experience, number of GPs working in the practice, number of patients, region in which GP practices, risk preferences, rational decision-making score and intuitive decision-making score. The full models are presented in the S7 Table. However, this increase in perceived difficulty did not correspond to a higher level of decisional effort in either of the two case vignettes (see Fig 4).

The results in Table 4 demonstrate that the experimental manipulation did not exert an impact on decisional effort.

**Table 4:** Ordered logistic regression on perceived decision effort [1;5].

	Sleeping tablets					Antibiotics			
	Unadjusted model		Adjusted model^a^		Unadjusted model			Adjusted model^a^	
	OR	95% CI	aOR	95% CI	OR		95% CI	aOR	95% CI
Overall									
Condition									
Control	Ref.		Ref.		Ref.			Ref.	
Fellow GP	1.369	0.901–2.079	1.349	0.871–2.090	0.987		0.650–1.499	1.066	0.688–1.651
Specialist	0.626	0.414–0.947*	0.666	0.434–1.023	0.768		0.515–1.147	0.732	0.481–1.113
N	475		475		475			475	

* *p*<0.05

** *p*<0.01.

a Covariates included in the adjusted models are GP’s age, gender, work experience, number of GPs working in the practice, number of patients, region in which GP practices, risk preferences, rational decision-making score and intuitive decision-making score. The full models are presented in the S8 Table.

## Discussion

This study represents the first experimental investigation into herding-like behaviour among GPs. Utilizing a web-based survey with case vignettes adapted from previous literature, we have demonstrated that the prescription decisions of GPs regarding sleeping tables and antibiotics may be influenced by recommendations. Notably, GPs were more willing to prescribe when receiving advice from a specialist. Conversely, a similar recommendation from a fellow GP influenced prescription decisions only in the sleeping tablet scenario, where GPs were less inclined to prescribe it. The results of this study partially validate earlier observational research which indicated that recommendations from other healthcare professionals can lead GPs into making medical errors [15,21,22]. While our research found evidence suggesting that herding-like behaviour could lead to prescription errors and in some cases protect against them, it offered limited insight into the mechanisms and motivations behind this phenomenon. Survey participants indicated that the decision whether to prescribe antibiotics became more challenging when they received recommendations to do so from a fellow GP or specialist. While this observation suggests that the introduction of additional information may have caused some confusion, the results do not indicate an increase in cognitive effort. The different frequency of choice of the control condition between the two case vignettes might be attributed to the GPs’ awareness of antibiotic resistance. GPs may be more reluctant to prescribe antibiotics due to their concerns about the broader issue of antimicrobial resistance [36]. Possible explanations for the influence of the specialists’ recommendation, but not the recommendation from the fellow GP, could include reputation effects or asymmetric information effects [37–39]. The reputation effect is rooted in injunctive social norms, wherein individuals are influenced by the behaviour or opinions of those with high reputation or perceived status [27]. In our study, GPs might have adhered to specialists’ recommendations due to specialists’ status as opinion leaders and their standing as reputable, successful, or credible figures in the medical field. Studies investigating stock market behaviour found a positive correlation between the reputation and credibility of forecasts and herding-like behaviour [39]. Furthermore, the asymmetric information effect suggests that GPs assume specialists possessed more comprehensive information when making the recommendations [37,38]. Asymmetric information can create situations where GPs fear making the wrong decision, particularly if their peers seem to have better information. To avoid potential negative outcomes, they might follow the recommendation of those who appear to be better informed. Future studies could attempt to decipher the mechanism behind the observed herding-like behaviour by conducting a similar experiment, but in which the fellow GP condition is substituted with an alternative specialist condition, incorporating an additional sentence clarifying that the specialist had access to the same information when making the recommendation. The finding that GPs do not follow or even contradict the recommendations of fellow GPs may imply a lack of trust. Prior literature on clinicians’ trust in their colleagues indicates that this trust is influenced not only by perceived competence but also by contracts and communication [40]. Future studies should incorporate trust measures as potential explanatory variables for herding-like behaviour.

The study has several limitations. Firstly, it featured a web-based experimental approach with hypothetical case vignettes and treatment intentions as outcomes [41], lacking behavioural validation in the real-world setting. Secondly, our study did not contain any questions about the perception of the recommendations or their source. Thirdly, there could have been limited contextual Information: The web-based format might not have provided GPs with the full clinical context, which could impact their prescription decisions and the study’s conclusions.

Our findings have practical ethical and legal implications as they confirmed that social information may be a possible underlying cause for medical errors leading to complications (i.e. a wrong recommendation by a physician could trigger a series of false diagnostic and treatment decisions of other physicians) [42,43]. In order to prevent these with educational interventions, future research is needed to understand the underlying mechanisms and motivations.

## Conclusion

This is the first experimental study providing evidence that herding-like behaviour among GPs may result in prescription errors. The findings indicate that GPs are more inclined to prescribe medication that diverges from best practice clinical guidelines when they receive comparable recommendations from specialists. As our study only investigated hypothetical scenarios, subsequent studies should explore herding-like behaviour in real-world settings.

## Supporting information

S1 Table Description of study sample in first case vignette (N = 475). (DOCX)

S2 Table Description of study sample in second case vignette (N = 475). (DOCX)

S3 Table Binary logistic regression on prescribing sleeping tablets in case vignette 1 (N = 475). (DOCX)

S4 Table Binary logistic regression on prescribing antibiotics in case vignette 2 (N = 475). (DOCX)

S5 Table Binary logistic regression with interaction terms on prescribing sleeping tablets in case vignette 1 (N = 475). (DOCX)

S6 Table Binary logistic regression with interaction terms on prescribing antibiotics in case vignette 2 (N = 475). (DOCX)

S7 Table Ordered logistic regression on perceived decision difficulty (N = 475). (DOCX)

S8 Table Ordered logistic regression on cognitive effort (N = 475). (DOCX)

S1 Fig Screenshots of the sleeping table case vignette. (DOCX)

S2 Fig Screenshots of the antibiotics case vignette. (DOCX)

S3 Fig Distribution of risk preferences [0;10]. (DOCX)

S4 Fig Distribution of rational decision-making score [5;25]. (DOCX)

S5 Fig Distribution of intuitive decision-making score [5;25]. (DOCX)

S6 Fig Individual prescription decisions in the two case vignettes. (DOCX)
