# Supplementary material for: Herding-like behaviour in medical decision making: An experimental study investigating general practitioners’ prescription behaviour
Source: PLoS One. 2024 Jul 8;19(7):e0297019. doi: 10.1371/journal.pone.0297019 (PMC11230524; doi:10.1371/journal.pone.0297019)
Supplement: S2 Table — (DOCX) [file pone.0297019.s002.docx]

**S2 Table.** Description of study sample in second case vignette (N=475)

|  | Control  (N=170) | | Fellow GP  (N=138) | | Specialist  (N=167) | | Total  (N=475) | |
| --- | --- | --- | --- | --- | --- | --- | --- | --- |
| Variable |  |  |  |  |  |  | N | (%) |
| Age |  |  |  |  |  |  |  |  |
| Below 30 | 2 | (1.2) | 2 | (14.5) | 0 | (0.0) | 4 | (0.8) |
| Between 30 and 39 | 87 | (51.2) | 53 | (38.4) | 65 | (38.9) | 205 | (43.2) |
| Between 40 and 49 | 49 | (28.8) | 56 | (40.6) | 73 | (43.7) | 178 | (37.5) |
| Between 50 and 59 | 23 | (13.5) | 18 | (13.0) | 23 | (13.8) | 64 | (13.5) |
| 60 or older | 9 | (5.3) | 8 | (6.5) | 6 | (3.6) | 24 | (5.0) |
| Gender |  |  |  |  |  |  |  |  |
| Female | 87 | (51.2) | 75 | (54.3) | 95 | (56.9) | 257 | (54.1) |
| Male | 82 | (48.2) | 60 | (43.5) | 71 | (42.5) | 213 | (44.8) |
| Other | 1 | (0.6) | 3 | (2.2) | 1 | (0.6) | 5 | (1.1) |
| Work experience |  |  |  |  |  |  |  |  |
| Between 2 and 5 years | 39 | (22.9) | 33 | (23.9) | 33 | (19.8) | 105 | (22.1) |
| Between 6 and 10 years | 56 | (32.9) | 36 | (26.1) | 44 | (26.3) | 136 | (28.6) |
| Between 11 and 20 years | 52 | (30.6) | 43 | (31.2) | 68 | (40.7) | 163 | (34.3) |
| More than 20 years | 23 | (13.5) | 26 | (18.8) | 22 | (13.2) | 71 | (15.0) |
| Number of GPs working in the practice | | | |  |  |  |  |  |
| Just me | 0 | (0.0) | 1 | (0.7) | 2 | (1.2) | 3 | (0.6) |
| Between 2 and 5 | 57 | (33.5) | 60 | (43.5) | 53 | (31.7) | 179 | (35.8) |
| Between 6 and 10 | 68 | (40.0) | 51 | (37.0) | 79 | (47.3) | 198 | (41.7) |
| More than 10 | 45 | (26.5) | 26 | (18.8) | 33 | (19.8) | 104 | (21.9) |
| Number of patients registered in the practice | | | |  |  |  |  |  |
| Up to 1000 | 3 | (1.8) | 5 | (3.6) | 2 | (1.2) | 10 | (2.1) |
| Between 1001 and 5000 | 8 | (4.7) | 15 | (10.9) | 21 | (12.6) | 44 | (9.3) |
| Between 5001 and 10000 | 56 | (32.9) | 50 | (36.2) | 58 | (34.7) | 164 | (34.5) |
| More than 10000 | 103 | (60.6) | 68 | (49.3) | 86 | (51.5) | 257 | (54.1) |
| Region in which GP practices | | | |  |  |  |  |  |
| London | 34 | (21.0) | 47 | (30.7) | 29 | (18.1) | 110 | (23.2) |
| West Midlands | 19 | (11.7) | 19 | (12.4) | 21 | (13.1) | 59 | (12.4) |
| East Midlands | 20 | (12.3) | 12 | (7.8) | 17 | (10.6) | 49 | (10.3) |
| South West | 15 | (9.3) | 15 | (9.8) | 15 | (9.4) | 45 | (9.5) |
| South East | 21 | (13.0) | 20 | (13.1) | 37 | (23.1) | 78 | (16.4) |
| Yorkshire and the Humber | 16 | (9.9) | 12 | (7.8) | 15 | (9.4) | 43 | (9.0) |
| North West | 7 | (4.3) | 6 | (3.9) | 6 | (3.8) | 19 | (4.0) |
| North East | 30 | (18.5) | 22 | (14.4) | 20 | (12.5) | 72 | 15.2) |
| Risk preference [1;10] – Mean and standard deviation | 4.55 | (1.98) | 4.05 | (2.04) | 4.35 | (1.95) | 4.34 | (2.00) |
| Rational decision making [5;25] – Mean and standard deviation | 20.92 | (2.78) | 21.28 | (2.51) | 21.19 | (2.23) | 21.12 | (2.52) |
| Intuitive decision making [5;25] – Mean and standard deviation | 14.14 | (3.36) | 14.05 | (3.09) | 14.52 | (3.24) | 14.25 | (3.24) |
