# Supplementary material for: Herding-like behaviour in medical decision making: An experimental study investigating general practitioners’ prescription behaviour
Source: PLoS One. 2024 Jul 8;19(7):e0297019. doi: 10.1371/journal.pone.0297019 (PMC11230524; doi:10.1371/journal.pone.0297019)
Supplement: S3 Table — (DOCX) [file pone.0297019.s003.docx]

**S3 Table.** Binary logistic regression on prescribing sleeping tablets in case vignette 1 (N=475)

|  |  | Unadjusted model | |  | Adjusted model | |
| --- | --- | --- | --- | --- | --- | --- |
|  | (%) | OR | 95% CI |  | aOR | 95% CI |
| Overall | (55.0) |  |  |  |  |  |
| Condition |  |  |  |  |  |  |
| Control | (55.6) | Ref. |  |  | Ref. |  |
| Fellow GP | (36.6) | 0.424 | 0.269 - 0.668** |  | 0.416 | 0.256 - 0.676** |
| Specialist | (73.8) | 2.248 | 1.406 - 3.593** |  | 2.168 | 1.325 - 3.546** |
| Age |  |  |  |  |  |  |
| Up to 39 | (53.6) | Ref. |  |  | Ref. |  |
| Between 40 and 49 | (55.6) | 1.085 | 0.726 - 1.622 |  | 0.869 | 0.485 - 1.556 |
| Between 50 and 59 | (57.8) | 1.187 | 0.674 - 2.090 |  | 0.985 | 0.404 - 2.405 |
| 60 or older | (54.2) | 1.024 | 0.438 - 2.389 |  | 1.045 | 0.277 - 3.947 |
| Gender |  |  |  |  |  |  |
| Male | (55.3) | Ref. |  |  | Ref. |  |
| Female | (43.0) | 0.950 | 0.660 - 1.369 |  | 0.868 | 0.573 - 1.316 |
| Other | (80.0) | 3.239 | 0.357 - 29.384 |  | 3.150 | 0.315 - 31.537 |
| Work experience | | | |  |  |  |
| Between 2 and 5 years | (45.7) | Ref. |  |  | Ref. |  |
| Between 6 and 10 years | (59.6) | 1.749 | 1.045 - 2.925* |  | 1.798 | 1.008 - 3.209* |
| Between 11 and 20 years | (57.7) | 1.618 | 0.987 - 2.651 |  | 1.925 | 0.954 - 3.886 |
| More than 20 years | (53.5) | 1.367 | 0.747 - 2.502 |  | 1.590 | 0.570 - 4.432 |
| Number of GPs working in practice | | | | | | |
| Just me | (66.7) | Ref. |  |  | Ref. |  |
| Between 2 and 5 | (54.1) | 0.590 | 0.052 - 6.628 |  | 0.930 | 0.066 - 13.077 |
| Between 6 and 10 | (54.0) | 0.588 | 0.052 - 6.590 |  | 0.934 | 0.064 - 13.677 |
| More than 10 | (57.7) | 0.682 | 0.060 - 7.758 |  | 0.877 | 0.057 - 13.387 |
| Number of patients registered in the practice | | | | | | |
| Up to 1000 | (50.0) | Ref. |  |  | Ref. |  |
| Between 1001 and 5000 | (61.4) | 1.588 | 0.400 - 6.314 |  | 1.002 | 0.208 - 4.826 |
| Between 5001 and 10000 | (48.8) | 0.952 | 0.266 - 3.415 |  | 0.840 | 0.190 - 3.709 |
| More than 10000 | (58.0) | 1.380 | 0.390 - 4.884 |  | 0.946 | 0.206 - 4.355 |
| Region in which GP practises | | | |  |  |  |
| London | (48.2) | Ref. |  |  | Ref. |  |
| West Midlands | (55.9) | 1.365 | 0.723 - 2.577 |  | 1.361 | 0.679 - 2.728 |
| East Midlands | (63.3) | 1.852 | 0.928 - 3.696 |  | 1.654 | 0.778 - 3.514 |
| South West | (55.6) | 1.344 | 0.670 - 2.699 |  | 1.311 | 0.611 - 2.812 |
| South East | (62.8) | 1.817 | 1.005 - 3.285* |  | 1.518 | 0.790 - 2.915 |
| Yorkshire and the Humber | (67.4) | 2.228 | 1.064 - 4.666* |  | 1.927 | 0.862 - 4.308 |
| North West | (31.6) | 0.496 | 0.176 - 1.400 |  | 0.447 | 0.143 - 1.394 |
| North East | (48.6) | 1.017 | 0.561 - 1.844 |  | 0.923 | 0.481 - 1.768 |
| Risk preference [1;10] |  | 1.028 | 0.939 - 1.126 |  | 1.013 | 0.914 - 1.123 |
| Rational decision making [5;25] |  | 0.985 | 0.917 - 1.059 |  | 0.994 | 0.919 - 1.076 |
| Intuitive decision making [5;25] |  | 1.082 | 1.022 - 1.146** |  | 1.087 | 1.020 - 1.158** |
| N |  | 475 |  |  | 475 |  |

* *p*<0.05; ** *p*<0.01
