# Supplementary material for: Herding-like behaviour in medical decision making: An experimental study investigating general practitioners’ prescription behaviour
Source: PLoS One. 2024 Jul 8;19(7):e0297019. doi: 10.1371/journal.pone.0297019 (PMC11230524; doi:10.1371/journal.pone.0297019)
Supplement: S4 Table — (DOCX) [file pone.0297019.s004.docx]

**S4 Table.** Binary logistic regression on prescribing antibiotics in case vignette 2 (N=475)

|  |  | Unadjusted model | |  | Adjusted model | |
| --- | --- | --- | --- | --- | --- | --- |
|  | (%) | OR | 95% CI |  | aOR | 95% CI |
| Overall | (15.8) |  |  |  |  |  |
| Condition |  |  |  |  |  |  |
| Control | (12.4) | Ref. |  |  | Ref. |  |
| Fellow GP | (10.1) | 0.801 | 0.391 - 1.641 |  | 0.885 | 0.417 - 1.881 |
| Specialist | (24.0) | 2.235 | 1.253 - 3.986** |  | 2.409 | 1.298 - 4.472** |
| Age |  |  |  |  |  |  |
| Up to 39 | (12.9) | Ref. |  |  | Ref. |  |
| Between 40 and 49 | (15.7) | 1.258 | 0.711 - 2.227 |  | 1.023 | 0.468 - 2.235 |
| Between 50 and 59 | (23.4) | 2.063 | 1.019 - 4.179* |  | 1.607 | 0.541 - 4.777 |
| 60 or older | (20.8) | 1.774 | 0.612 - 5.145 |  | 1.847 | 0.358 - 9.533 |
| Gender |  |  |  |  |  |  |
| Male | (14.0) | Ref. |  |  | Ref. |  |
| Female | (17.8) | 1.333 | 0.811 - 2.191 |  | 1.236 | 0.711 - 2.151 |
| Other | (20.0) | 1.535 | 0.167 - 14.123 |  | 2.337 | 0.227 - 24.089 |
| Work experience |  |  |  |  |  |  |
| Between 2 and 5 years | (12.4) | Ref. |  |  | Ref. |  |
| Between 6 and 10 years | (13.2) | 1.080 | 0.503 - 2.317 |  | 1.138 | 0.499 - 2.597 |
| Between 11 and 20 years | (18.4) | 1.596 | 0.790 - 3.224 |  | 1.428 | 0.551 - 3.696 |
| More than 20 years | (19.7) | 1.738 | 0.762 - 3.963 |  | 1.117 | 0.294 - 4.247 |
| Number of GPs working in practice | | | | | | |
| Up to 5 | (16.8) | Ref. |  |  | Ref. |  |
| More than 5 | (15.2) | 0.892 | 0.537 - 1.482 |  | 0.825 | 0.456 - 1.495 |
| Number of patients registered in the practice | | | | | | |
| Up to 5000 | (14.8) | Ref. |  |  | Ref. |  |
| More than 5000 | (15.9) | 1.088 | 0.491 – 2.410 |  | 1.586 | 0.624 - 4.035 |
| Region in which GP practises | | | | | | |
| London | (17.3) | Ref. |  |  | Ref. |  |
| West Midlands | (13.6) | 0.751 | 0.307 - 1.838 |  | 0.926 | 0.360 - 2.381 |
| East Midlands | (12.2) | 0.668 | 0.249 - 1.793 |  | 0.663 | 0.234 - 1.877 |
| South West | (13.3) | 0.737 | 0.273 - 1.986 |  | 0.643 | 0.215 - 1.926 |
| South East | (19.2) | 1.140 | 0.539 - 2.412 |  | 1.084 | 0.483 - 2.434 |
| Yorkshire and the Humber | (23.3) | 1.451 | 0.612 - 3.440 |  | 1.363 | 0.530 - 3.505 |
| North East and East | (12.1) | 0.659 | 0.296 - 1.467 |  | 0.689 | 0.293 - 1.618 |
| Risk preference [1;10] |  | 1.073 | 0.946 - 1.216 |  | 1.002 | 0.876 - 1.147 |
| Rational decision making [5;25] |  | 0.911 | 0.832 - 0.997* |  | 0.912 | 0.827 - 1.005 |
| Intuitive decision making [5;25] |  | 1.183 | 1.093 - 1.280** |  | 1.170 | 1.078 - 1.271** |
| N |  | 475 |  |  | 475 |  |

* *p*<0.05; ** *p*<0.01
