# Supplementary material for: Herding-like behaviour in medical decision making: An experimental study investigating general practitioners’ prescription behaviour
Source: PLoS One. 2024 Jul 8;19(7):e0297019. doi: 10.1371/journal.pone.0297019 (PMC11230524; doi:10.1371/journal.pone.0297019)
Supplement: S5 Table — (DOCX) [file pone.0297019.s005.docx]

**S5 Table.** Binary logistic regression with interaction terms on prescribing sleeping tablets in case vignette 1 (N=475)

|  |  | Unadjusted model | |  | Adjusted model | |
| --- | --- | --- | --- | --- | --- | --- |
|  | (%) | OR | 95% CI |  | aOR | 95% CI |
| Overall | (55.0) |  |  |  |  |  |
| Condition and work experience |  |  |  |  |  |  |
| Control – up to 10 years | (55.8) | Ref. |  |  | Ref. |  |
| Fellow GP – up to 10 years | (30.6) | 0.348 | 0.180 - 0.672** |  | 0.349 | 0.176 - 0.691** |
| Specialist – up to 10 years | (71.1) | 1.946 | 1.029 - 3.681* |  | 2.057 | 1.061 - 3.989* |
| Control – more than 10 years | (55.3) | 0.978 | 0.526 - 1.820 |  | 1.104 | 0.500 - 2.440 |
| Fellow GP – more than 10 years | (38.3) | 0.491 | 0.265 - 0.911* |  | 0.506 | 0.232 - 1.106 |
| Specialist – more than 10 years | (76.6) | 2.595 | 1.317 - 5.112** |  | 2.470 | 1.091 - 5.594* |
| Age |  |  |  |  |  |  |
| Up to 39 | (53.6) | Ref. |  |  | Ref. |  |
| Between 40 and 49 | (55.6) | 1.085 | 0.726 - 1.622 |  | 1.012 | 0.574 - 1.783 |
| Between 50 and 59 | (57.8) | 1.187 | 0.674 - 2.090 |  | 1.053 | 0.478 - 2.321 |
| 60 or older | (54.2) | 1.024 | 0.438 - 2.389 |  | 1.036 | 0.359 - 2.988 |
| Gender |  |  |  |  |  |  |
| Male | (55.3) | Ref. |  |  | Ref. |  |
| Female | (43.0) | 0.950 | 0.660 - 1.369 |  | 0.861 | 0.571 - 1.299 |
| Other | (80.0) | 3.239 | 0.357 - 29.384 |  | 3.175 | 0.293 - 34.403 |
| Number of GPs working in practice | | | | | | |
| Up to 5 | (54.3) | Ref. |  |  | Ref. |  |
| More than 5 | (55.3) | 1.040 | 0.714 - 1.513 |  | 1.044 | 0.665 - 1.637 |
| Number of patients registered in the practice | | | | | | |
| Up to 5000 | (59.3) | Ref. |  |  | Ref. |  |
| More than 5000 | (54.4) | 0.820 | 0.461 - 1.458 |  | 0.853 | 0.430 - 1.693 |
| Region in which GP practises | | | |  |  |  |
| London | (48.2) | Ref. |  |  | Ref. |  |
| West Midlands | (55.9) | 1.365 | 0.723 - 2.577 |  | 1.320 | 0.659 - 2.643 |
| East Midlands | (63.3) | 1.852 | 0.928 - 3.696 |  | 1.677 | 0.795 - 3.536 |
| South West | (55.6) | 1.344 | 0.670 - 2.699 |  | 1.273 | 0.588 - 2.754 |
| South East | (62.8) | 1.817 | 1.005 - 3.285* |  | 1.475 | 0.769 - 2.832 |
| Yorkshire and the Humber | (67.4) | 2.228 | 1.064 - 4.666* |  | 1.937 | 0.873 - 4.298 |
| North West and North East | (45.1) | 0.882 | 0.505 - 1.539 |  | 0.813 | 0.445 - 1.486 |
| Risk preference [1;10] |  | 1.028 | 0.939 - 1.126 |  | 1.009 | 0.911 - 1.116 |
| Rational decision making [5;25] |  | 0.985 | 0.917 - 1.059 |  | 1.006 | 0.930 - 1.087 |
| Intuitive decision making [5;25] |  | 1.082 | 1.022 - 1.146** |  | 1.091 | 1.024 - 1.162** |
| N |  | 475 |  |  | 475 |  |

* *p*<0.05; ** *p*<0.01
