# Supplementary material for: Herding-like behaviour in medical decision making: An experimental study investigating general practitioners’ prescription behaviour
Source: PLoS One. 2024 Jul 8;19(7):e0297019. doi: 10.1371/journal.pone.0297019 (PMC11230524; doi:10.1371/journal.pone.0297019)
Supplement: S6 Table — (DOCX) [file pone.0297019.s006.docx]

**S6 Table.** Binary logistic regression with interaction terms on prescribing antibiotics in case vignette 2 (N=475)

|  |  | Unadjusted model | |  | Adjusted model | |
| --- | --- | --- | --- | --- | --- | --- |
|  | (%) | OR | 95% CI |  | aOR | 95% CI |
| Overall | (15.8) |  |  |  |  |  |
| Condition and work experience |  |  |  |  |  |  |
| Control – up to10 years | (13.7) | Ref. |  |  | Ref. |  |
| Fellow GP – up to 10 years | (5.8) | 0.388 | 0.121 - 1.247 |  | 0.444 | 0.133 - 1.481 |
| Specialist – up to 10 years | (18.2) | 1.402 | 0.615 - 3.193 |  | 1.525 | 0.642 - 3.620 |
| Control – more than 10 years | (10.7) | 0.753 | 0.295 - 1.924 |  | 0.604 | 0.190 - 1.926 |
| Fellow GP – more than 10 years | (14.5) | 1.069 | 0.439 - 2.603 |  | 0.931 | 0.306 - 2.838 |
| Specialist – more than 10 years | (28.9) | 2.563 | 1.221 - 5.380* |  | 2.369 | 0.900 - 6.236 |
| Age |  |  |  |  |  |  |
| Up to 39 | (12.9) | Ref. |  |  | Ref. |  |
| Between 40 and 49 | (15.7) | 1.258 | 0.711 - 2.227 |  | 1.093 | 0.501 - 2.385 |
| Between 50 and 59 | (23.4) | 2.063 | 1.019 - 4.179* |  | 1.631 | 0.591 - 4.502 |
| 60 or older | (20.8) | 1.774 | 0.612 - 5.145 |  | 1.626 | 0.418 - 6.322 |
| Gender |  |  |  |  |  |  |
| Male | (14.0) | Ref. |  |  | Ref. |  |
| Female | (17.8) | 1.333 | 0.811 - 2.191 |  | 1.208 | 0.695 - 2.098 |
| Other | (20.0) | 1.535 | 0.167 - 14.123 |  | 2.393 | 0.228 - 25.089 |
| Number of GPs working in practice | | | | | | |
| Up to 5 | (16.8) | Ref. |  |  | Ref. |  |
| More than 5 | (15.2) | 0.892 | 0.537 - 1.482 |  | 0.809 | 0.446 - 1.469 |
| Number of patients registered in the practice | | | | | | |
| Up to 5000 | (14.8) | Ref. |  |  | Ref. |  |
| More than 5000 | (15.9) | 1.088 | 0.491 - 2.410 |  | 1.612 | 0.625 - 4.156 |
| Region in which GP practises | | | | | | |
| London | (17.3) | Ref. |  |  | Ref. |  |
| West Midlands | (13.6) | 0.751 | 0.307 - 1.838 |  | 0.952 | 0.369 - 2.457 |
| East Midlands | (12.2) | 0.668 | 0.249 - 1.793 |  | 0.656 | 0.233 - 1.843 |
| South West | (13.3) | 0.737 | 0.273 - 1.986 |  | 0.616 | 0.205 - 1.852 |
| South East | (19.2) | 1.140 | 0.539 - 2.412 |  | 1.100 | 0.490 - 2.471 |
| Yorkshire and the Humber | (23.3) | 1.451 | 0.612 - 3.440 |  | 1.338 | 0.520 - 3.447 |
| North East and East | (12.1) | 0.659 | 0.296 - 1.467 |  | 0.690 | 0.294 - 1.619 |
| Risk preference [1;10] |  | 1.073 | 0.946 - 1.216 |  | 0.995 | 0.870 - 1.139 |
| Rational decision making [5;25] |  | 0.911 | 0.832 - 0.997* |  | 0.917 | 0.834 - 1.010 |
| Intuitive decision making [5;25] |  | 1.183 | 1.093 - 1.280** |  | 1.169 | 1.077 - 1.269** |
| N |  | 475 |  |  | 475 |  |

* *p*<0.05; ** *p*<0.01
