# Supplementary material for: Herding-like behaviour in medical decision making: An experimental study investigating general practitioners’ prescription behaviour
Source: PLoS One. 2024 Jul 8;19(7):e0297019. doi: 10.1371/journal.pone.0297019 (PMC11230524; doi:10.1371/journal.pone.0297019)
Supplement: S7 Table — (DOCX) [file pone.0297019.s007.docx]

**S7 Table.** Ordered logistic regression on perceived decision difficulty (N=475)

|  | Case vignette 1: Sleeping tablets | | | | |  | Case vignette 2: Antibiotics | | | | |  |
| --- | --- | --- | --- | --- | --- | --- | --- | --- | --- | --- | --- | --- |
|  | Unadjusted model | |  | Adjusted model | |  | Unadjusted model | |  | Adjusted model | | |
| Variable | OR | 95% CI |  | aOR | 95% CI |  | OR | 95% CI |  | aOR | 95% CI | |
| Overall |  |  |  |  |  |  |  |  |  |  |  | |
| Condition |  |  |  |  |  |  |  |  |  |  |  | |
| Control | Ref. |  |  | Ref. |  |  | Ref. |  |  | Ref. |  | |
| Fellow GP | 1.159 | 0.775 - 1.734 |  | 1.115 | 0.733 - 1.697 |  | 1.632 | 1.080 - 2.467* |  | 1.552 | 1.007 - 2.391* | |
| Specialist | 0.712 | 0.479 - 1.058 |  | 0.771 | 0.511 - 1.165 |  | 2.607 | 1.757 - 3.867** |  | 2.642 | 1.754 - 3.978** | |
| Age |  |  |  |  |  |  |  |  |  |  |  | |
| Up to 39 | Ref. |  |  | Ref. |  |  | Ref. |  |  | Ref. |  | |
| Between 40 and 49 | 1.047 | 0.727 - 1.507 |  | 0.924 | 0.568 - 1.503 |  | 1.081 | 0.753 - 1.551 |  | 1.135 | 0.702 - 1.836 | |
| Between 50 and 59 | 0.788 | 0.475 - 1.305 |  | 0.729 | 0.350 - 1.520 |  | 0.818 | 0.493 - 1.357 |  | 0.971 | 0.464 - 2.032 | |
| 60 or older | 0.663 | 0.310 - 1.414 |  | 0.676 | 0.223 - 2.046 |  | 0.911 | 0.432 - 1.919 |  | 1.538 | 0.513 - 4.606 | |
| Gender |  |  |  |  |  |  |  |  |  |  |  | |
| Male | Ref. |  |  | Ref. |  |  | Ref. |  |  | Ref. |  | |
| Female | 0.752 | 0.540 - 1.047 |  | 0.775 | 0.545 - 1.101 |  | 0.765 | 0.551 - 1.062 |  | 0.789 | 0.556 - 1.119 | |
| Other | 0.874 | 0.208 - 3.678 |  | 0.899 | 0.205 - 3.939 |  | 1.739 | 0.388 - 7.789 |  | 1.279 | 0.269 - 6.079 | |
| Work experience | | |  |  |  |  |  |  |  |  |  | |
| Between 2 and 5 years | Ref. |  |  | Ref. |  |  | Ref. |  |  | Ref. |  | |
| Between 6 and 10 years | 0.699 | 0.438 - 1.116 |  | 0.658 | 0.400 - 1.081 |  | 1.026 | 0.646 - 1.628 |  | 0.921 | 0.562 - 1.510 | |
| Between 11 and 20 years | 0.922 | 0.588 - 1.444 |  | 1.077 | 0.595 - 1.951 |  | 0.976 | 0.628 - 1.519 |  | 0.874 | 0.487 - 1.568 | |
| More than 20 years | 0.714 | 0.412 - 1.236 |  | 0.934 | 0.394 - 2.213 |  | 0.771 | 0.451 - 1.319 |  | 0.639 | 0.271 - 1.509 | |
| Number of GPs working in practice | | |  |  |  |  |  |  |  |  |  | |
| Just me | Ref. |  |  | Ref. |  |  | Ref. |  |  | Ref. |  | |
| Between 2 and 5 | 13.280 | 1.281 - 137.718* |  | 7.409 | 0.624 - 87.969 |  | 8.024 | 0.778 - 82.765 |  | 7.573 | 0.619 - 92.575 | |
| Between 6 and 10 | 13.797 | 1.333 - 142.835* |  | 7.591 | 0.619 - 93.104 |  | 7.125 | 0.693 - 73.293 |  | 5.977 | 0.475 - 75.231 | |
| More than 10 | 12.230 | 1.168 - 128.003* |  | 7.516 | 0.594 - 95.130 |  | 5.390 | 0.518 - 56.045 |  | 5.027 | 0.387 - 65.211 | |
| Number of patients registered in the practice | | |  |  |  |  |  |  |  |  |  | |
| Up to 1000 | Ref. |  |  | Ref. |  |  | Ref. |  |  | Ref. |  | |
| Between 1001 and 5000 | 2.983 | 0.821 - 10.830 |  | 3.420 | 0.907 - 12.892 |  | 4.024 | 1.128 - 14.353* |  | 3.548 | 0.916 - 13.744 | |
| Between 5001 and 10000 | 3.997 | 1.194 - 13.384* |  | 3.460 | 0.990 - 12.099 |  | 4.060 | 1.226 - 13.443* |  | 3.499 | 0.959 - 12.770 | |
| More than 10000 | 3.114 | 0.942 - 10.294 |  | 2.985 | 0.828 - 10.767 |  | 3.002 | 0.921 - 9.790 |  | 3.438 | 0.910 - 12.994 | |
| Region in which GP practises | | |  |  |  |  |  |  |  |  |  | |
| London | Ref. |  |  | Ref. |  |  | Ref. |  |  | Ref. |  | |
| West Midlands | 4.024 | 1.128 - 14.353* |  | 0.992 | 0.545 - 1.803 |  | 4.024 | 1.128 - 14.353* |  | 0.604 | 0.335 - 1.088 | |
| East Midlands | 4.060 | 1.226 - 13.443* |  | 1.166 | 0.623 - 2.183 |  | 4.060 | 1.226 - 13.443* |  | 1.072 | 0.569 - 2.022 | |
| South West | 3.002 | 0.921 - 9.790 |  | 1.444 | 0.759 - 2.747 |  | 3.002 | 0.921 - 9.790 |  | 0.714 | 0.375 - 1.358 | |
| South East | 4.024 | 1.128 - 14.353* |  | 0.738 | 0.425 - 1.282 |  | 4.024 | 1.128 - 14.353* |  | 0.893 | 0.523 - 1.528 | |
| Yorkshire and the Humber | 4.060 | 1.226 - 13.443* |  | 0.755 | 0.388 - 1.470 |  | 4.060 | 1.226 - 13.443* |  | 0.597 | 0.311 - 1.144 | |
| North West | 3.002 | 0.921 - 9.790 |  | 2.135 | 0.863 - 5.282 |  | 3.002 | 0.921 - 9.790 |  | 1.040 | 0.430 - 2.513 | |
| North East | 4.024 | 1.128 - 14.353* |  | 1.459 | 0.830 - 2.564 |  | 4.024 | 1.128 - 14.353* |  | 1.128 | 0.645 - 1.973 | |
| Risk preference [1;10] | 0.968 | 0.891 - 1.051 |  | 0.986 | 0.903 - 1.076 |  | 0.929 | 0.856 - 1.009 |  | 0.946 | 0.867 - 1.032 | |
| Rational decision making [5;25] | 1.008 | 0.946 - 1.073 |  | 1.010 | 0.945 - 1.079 |  | 1.001 | 0.938 - 1.067 |  | 0.999 | 0.934 - 1.068 | |
| Intuitive decision making [5;25] | 0.984 | 0.935 - 1.036 |  | 0.992 | 0.940 - 1.046 |  | 1.019 | 0.969 - 1.072 |  | 1.008 | 0.957 - 1.063 | |
| N | 475 |  |  | 475 |  |  | 475 |  |  | 475 |  | |

* *p*<0.05; ** *p*<0.01
