# Supplementary figures and images for: Herding-like behaviour in medical decision making: An experimental study investigating general practitioners’ prescription behaviour
Source: PLoS One. 2024 Jul 8;19(7):e0297019. doi: 10.1371/journal.pone.0297019 (PMC11230524; doi:10.1371/journal.pone.0297019)

## S1 Figure. Screenshots of the sleeping table case vignette

| 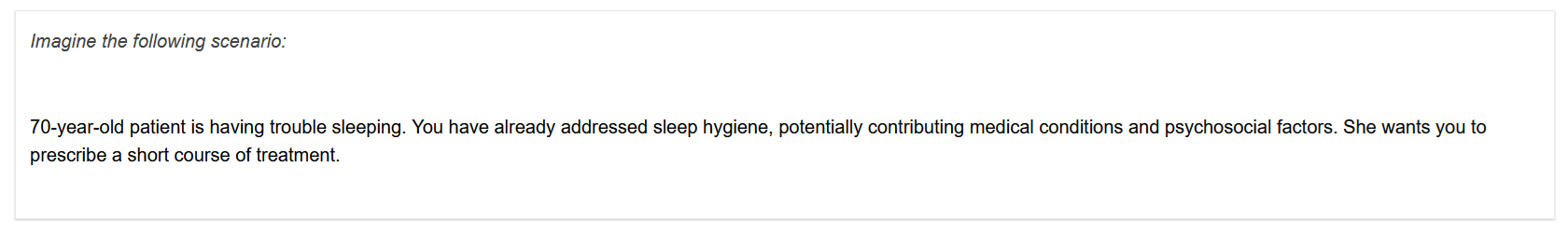 |
| --- |
| 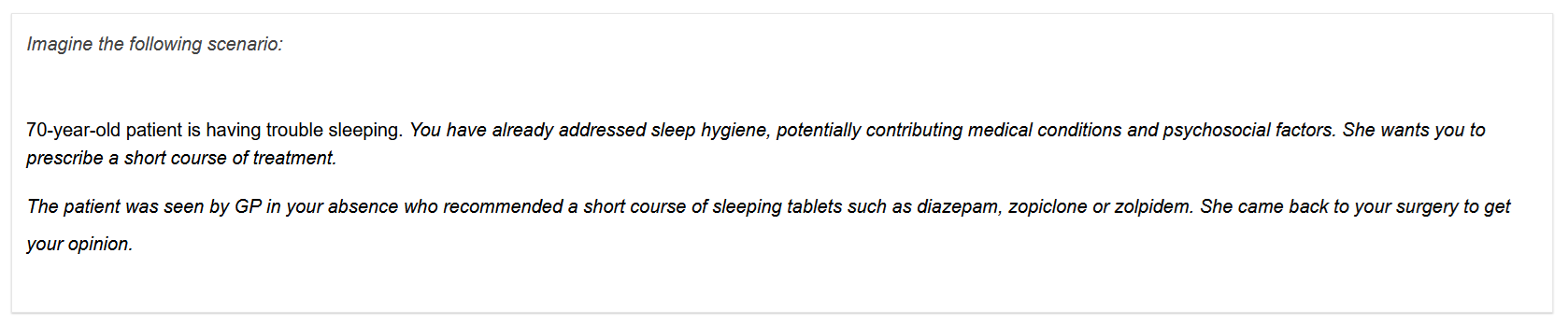 |
| 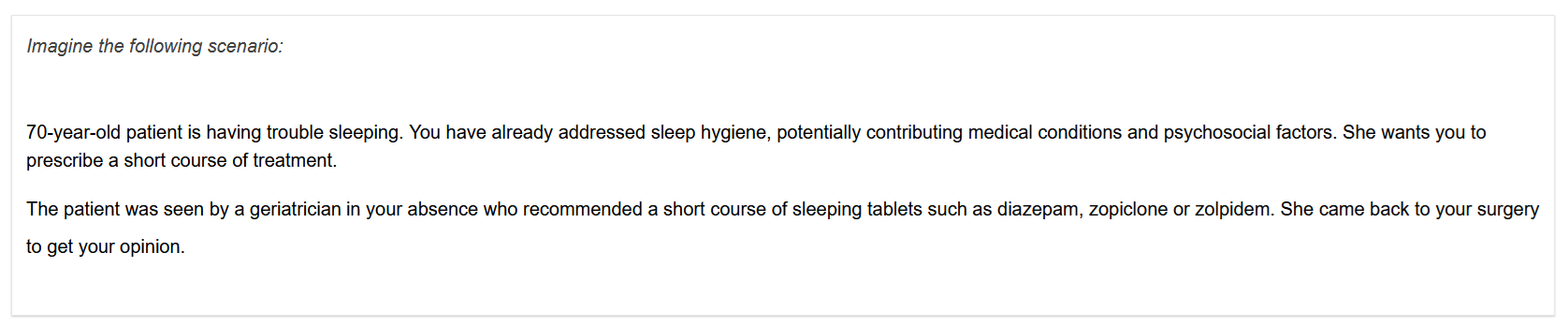 |

Supplement: S8 Table — (DOCX) [file pone.0297019.s008.docx]

## S1 Figure. Screenshots of the sleeping table case vignette

| 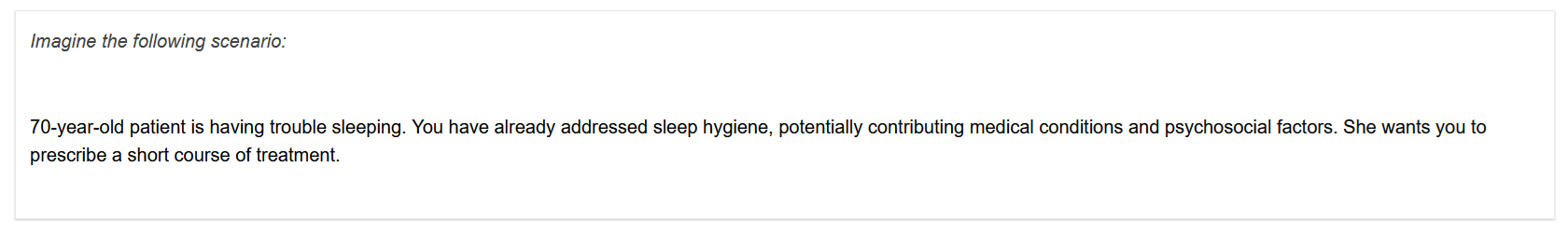 |
| --- |
| 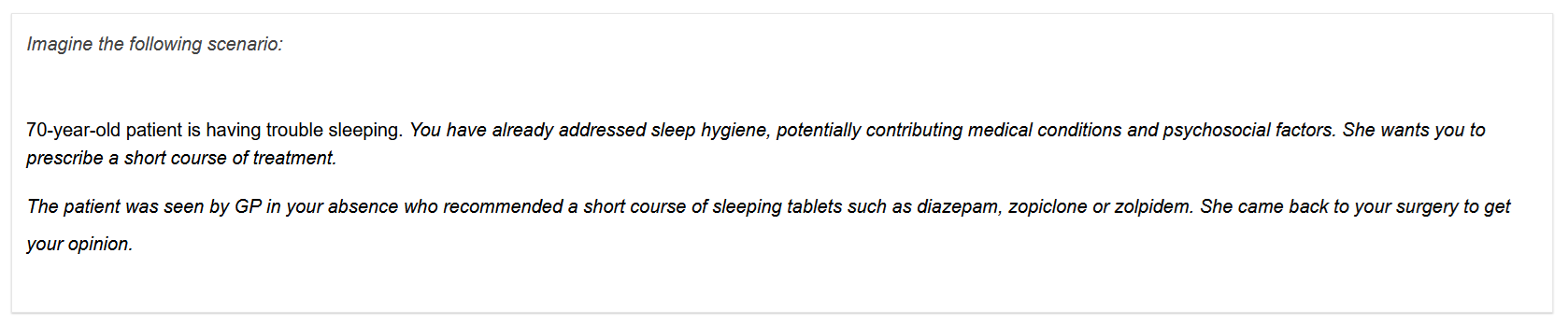 |
| 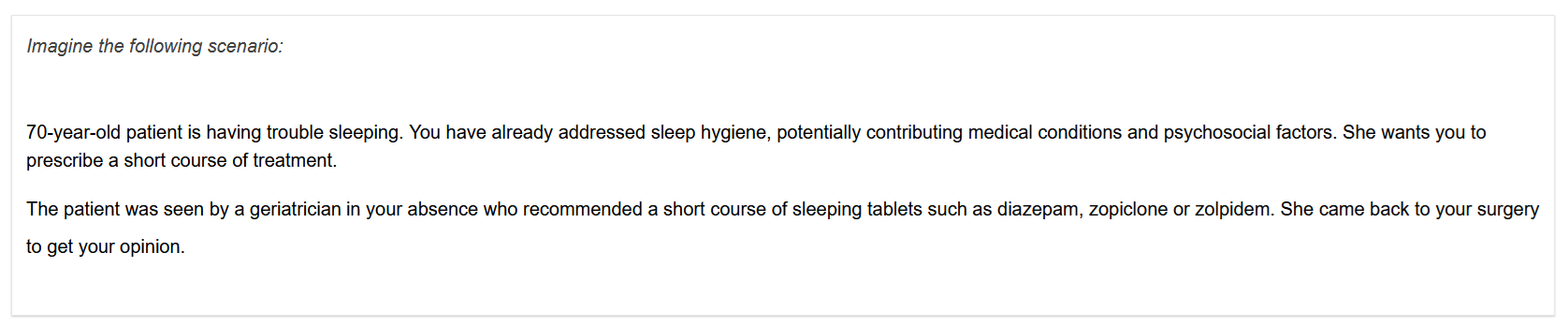 |

Supplement: S1 Fig — (DOCX) [file pone.0297019.s009.docx]

**S2 Figure.** Screenshots of the antibiotics case vignette

| 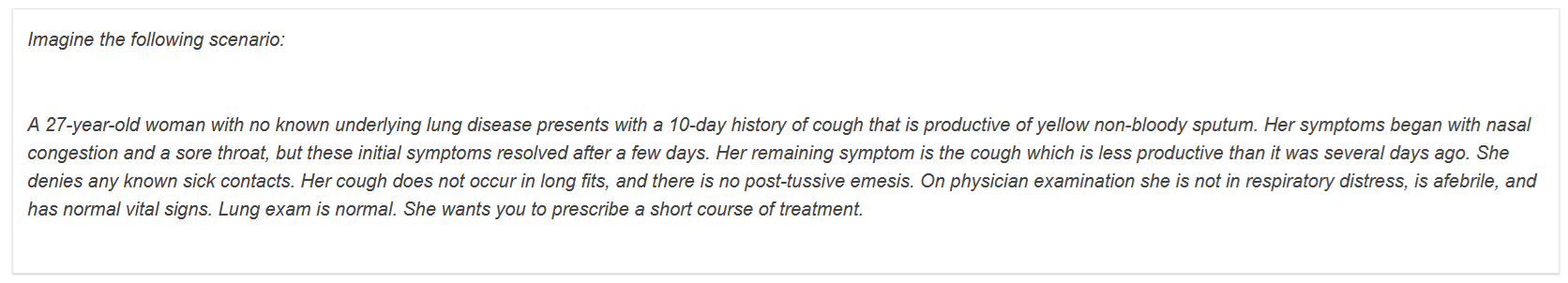 |
| --- |
| 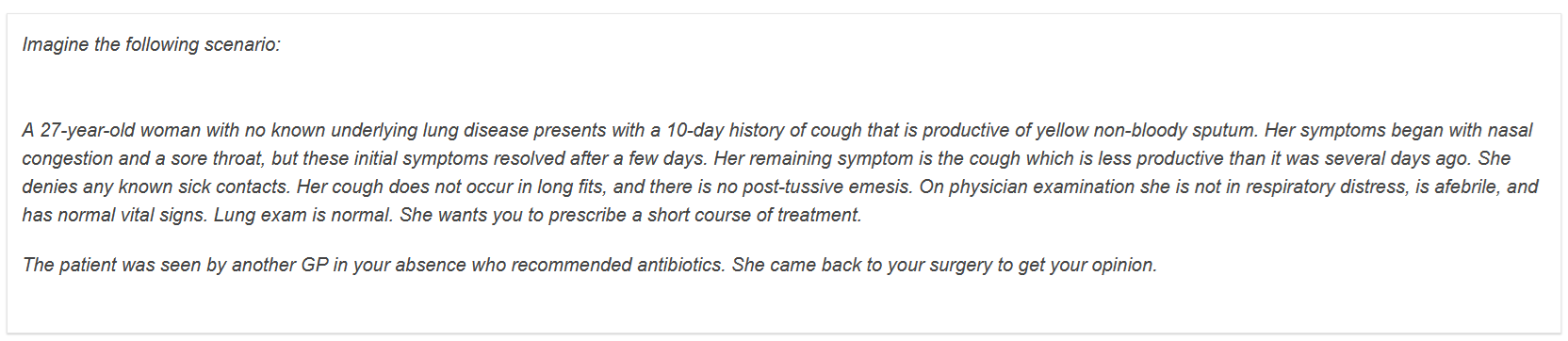 |
| 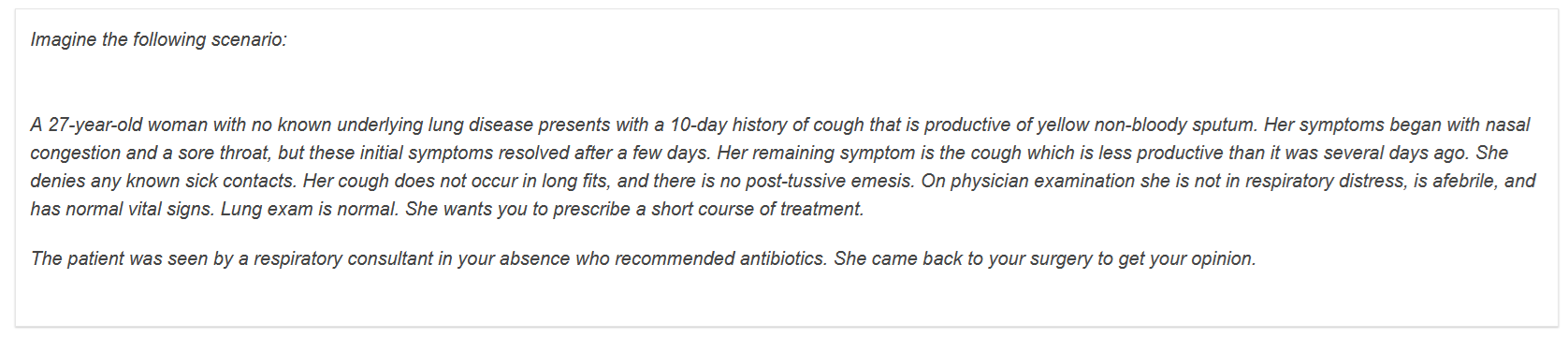 |

Supplement: S2 Fig — (DOCX) [file pone.0297019.s010.docx]

**S3 Figure.** Distribution of risk preferences [0;10]


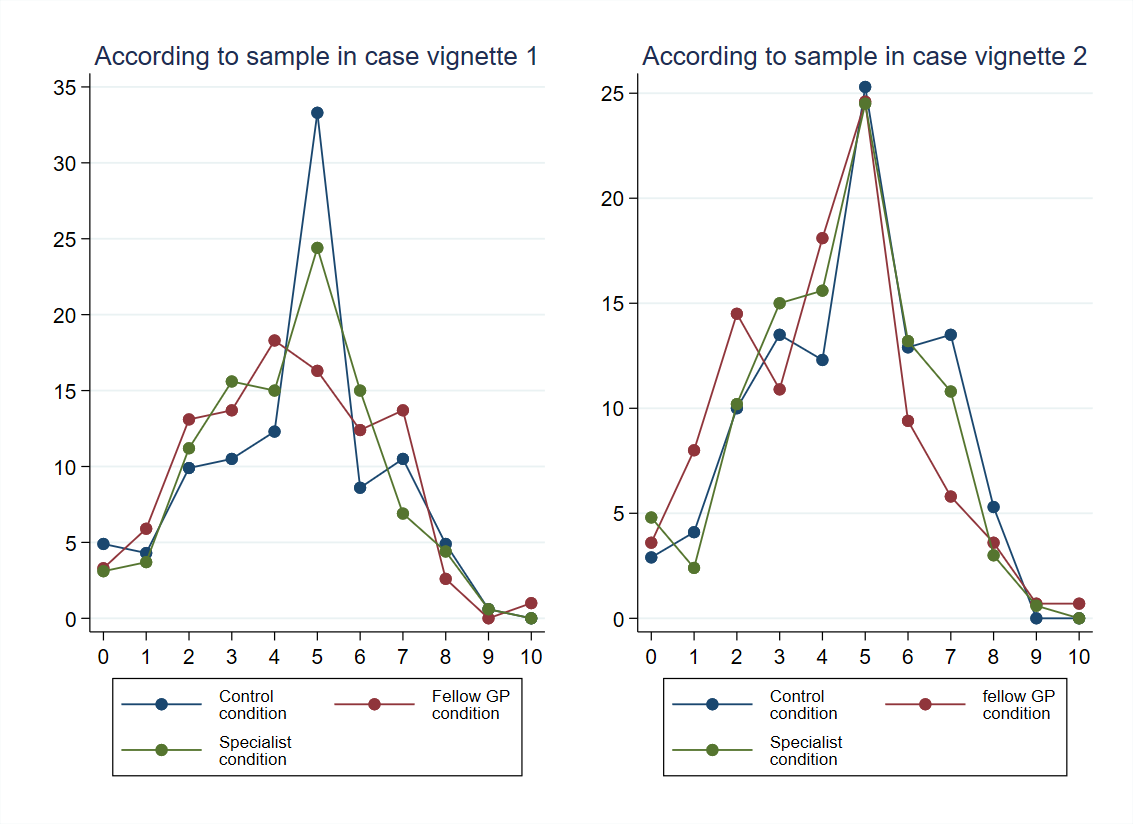

Supplement: S3 Fig — (DOCX) [file pone.0297019.s011.docx]

**S4 Figure.** Distribution of rational decision-making score [5;25]


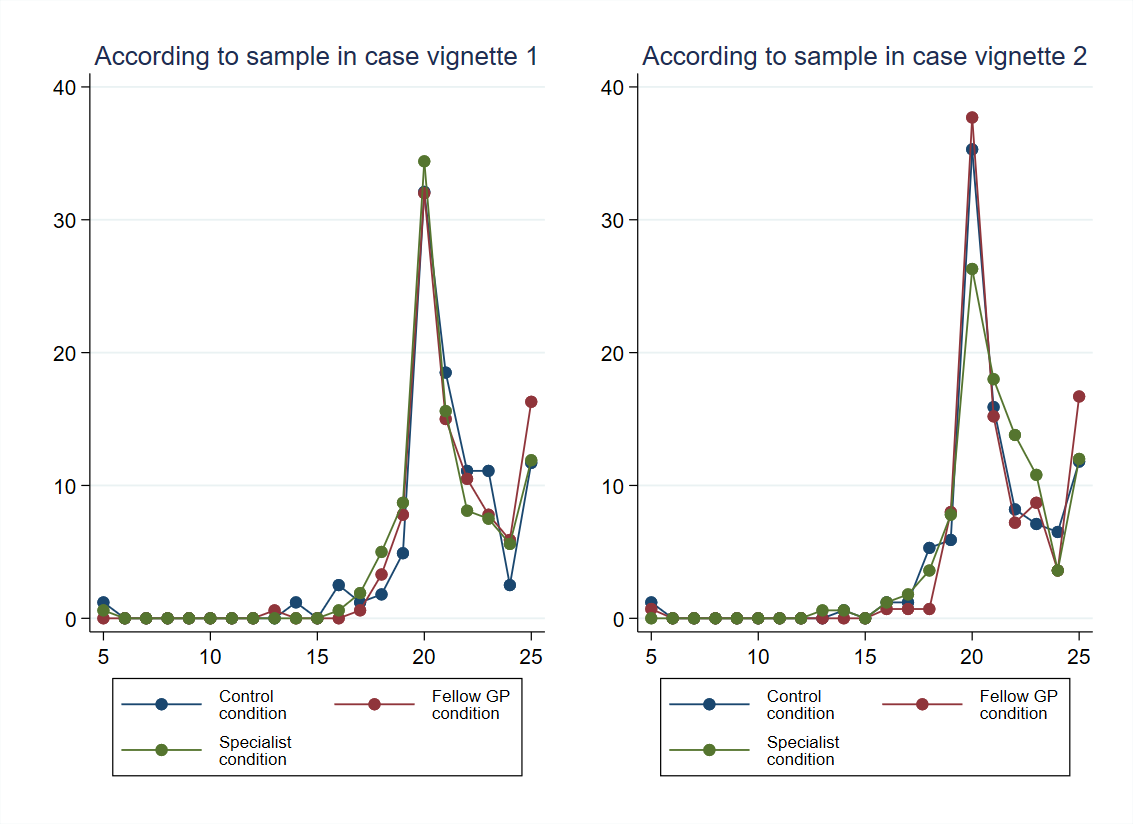

Supplement: S4 Fig — (DOCX) [file pone.0297019.s012.docx]

**S5 Figure**. Distribution of intuitive decision-making score [5;25]


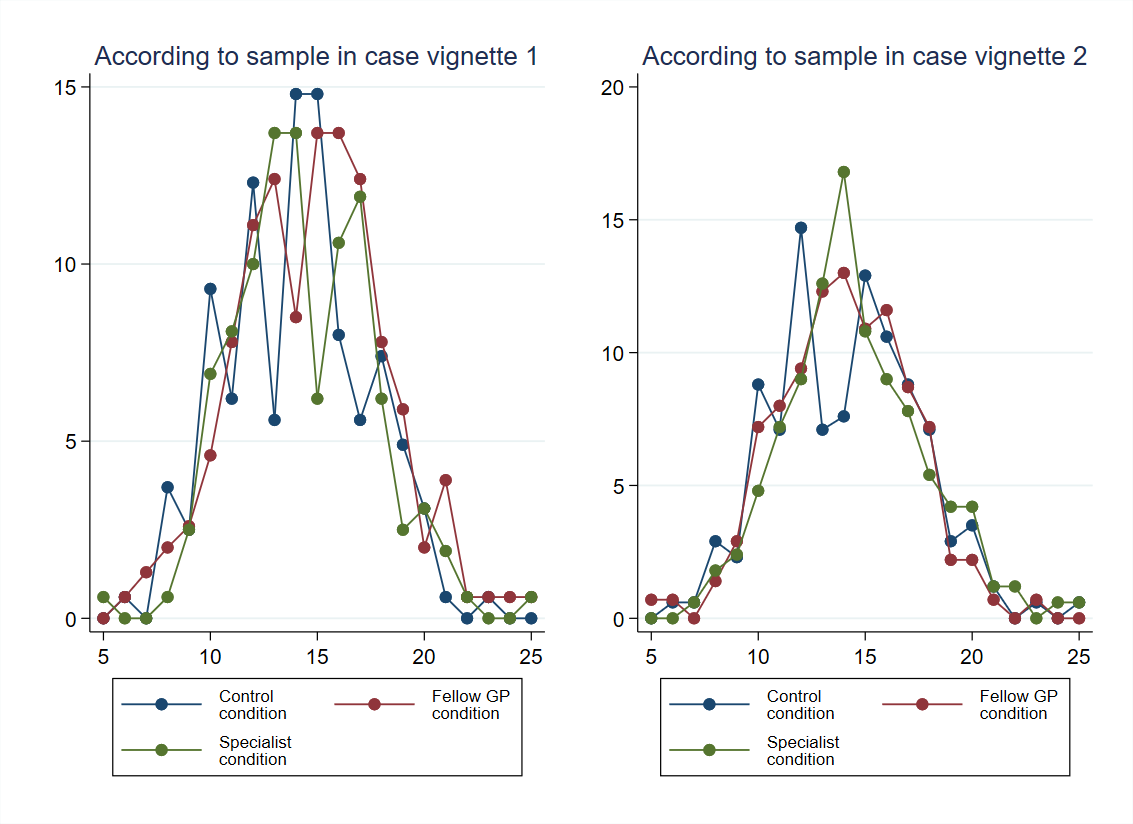

Supplement: S5 Fig — (DOCX) [file pone.0297019.s013.docx]

**S6 Figure.** Individual prescription decisions in the two case vignettes


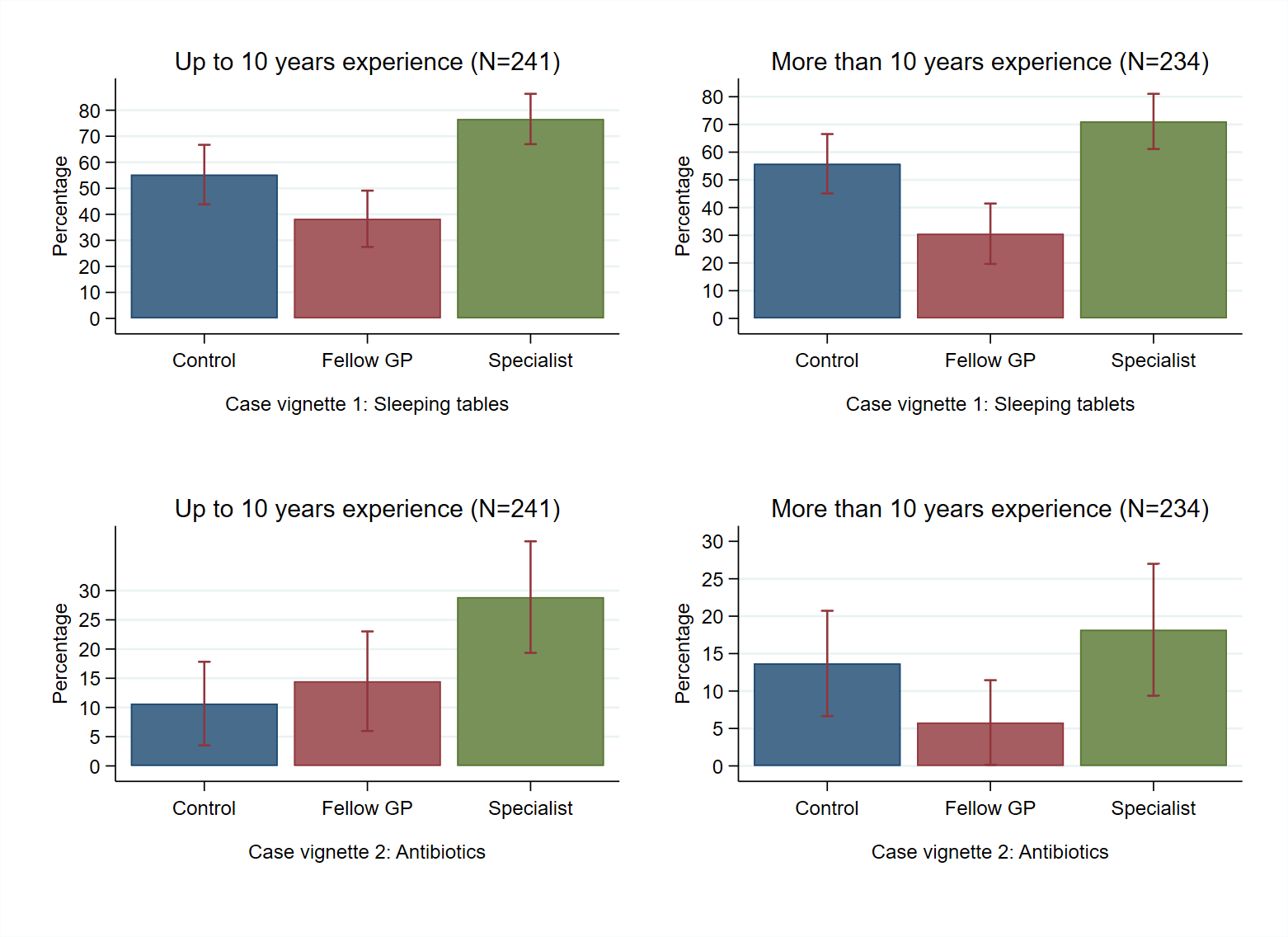

Supplement: S6 Fig — (DOCX) [file pone.0297019.s014.docx]
